# Supplementary material for: A conserved genetic interaction between Spt6 and Set2 regulates H3K36 methylation
Source: Nucleic Acids Res. 2019 Feb 22;47(8):3888–903. doi: 10.1093/nar/gkz119 (PMC6486648; doi:10.1093/nar/gkz119)
Supplement: Supplementary Data [file gkz119_supplemental_file.pdf]

Supplemental Figure S1

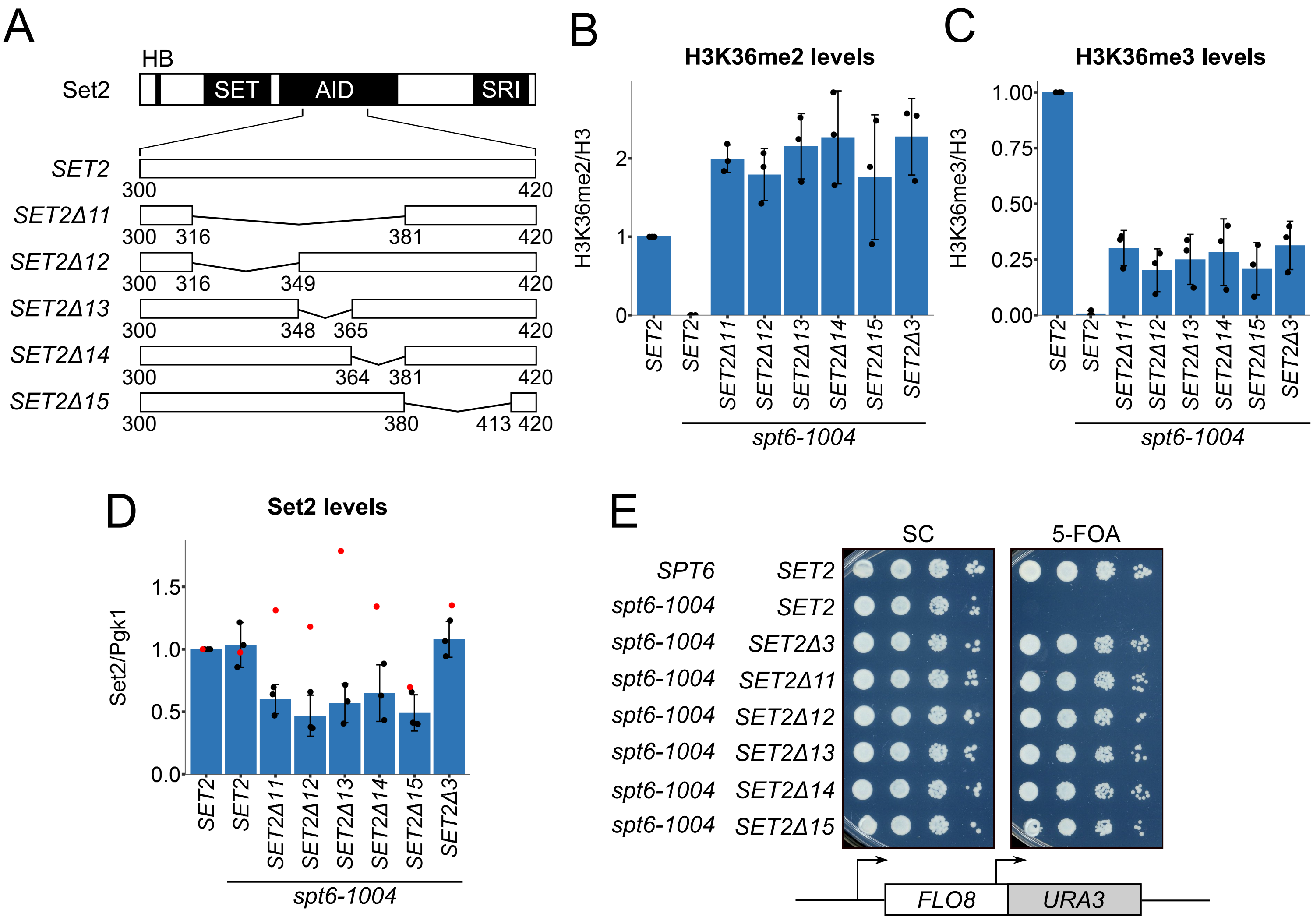

Supplemental Figure S1. Deletions in the region encoding the Set2 autoinhibitory domain partially rescue H3K36 methylation and intragenic transcription in *spt6-1004*. (**A**) The schematic depicts some of the domains in Set2 and the five mutants that have deletions in the region encoding the autoinhibitory domain. (**B-D**) Quantification of western blots assaying H3K36me2 (**B**), H3K36me3 (**C**), and Set2 (**D**) levels in *spt6-1004* strains with the indicated *SET2* mutations. The black dots represent the individual data points for three experiments and the bars show the mean +/- standard deviation. The red dots in (**D**) represent outlier values from one experiment which were not included in the calculation of the mean or error bars. (**E**) Spot tests of cells grown at 34°C assaying the effect of *SET2* mutations on expression of the *FLO8-URA3* reporter in an *spt6-1004* background.

Supplemental Figure S2

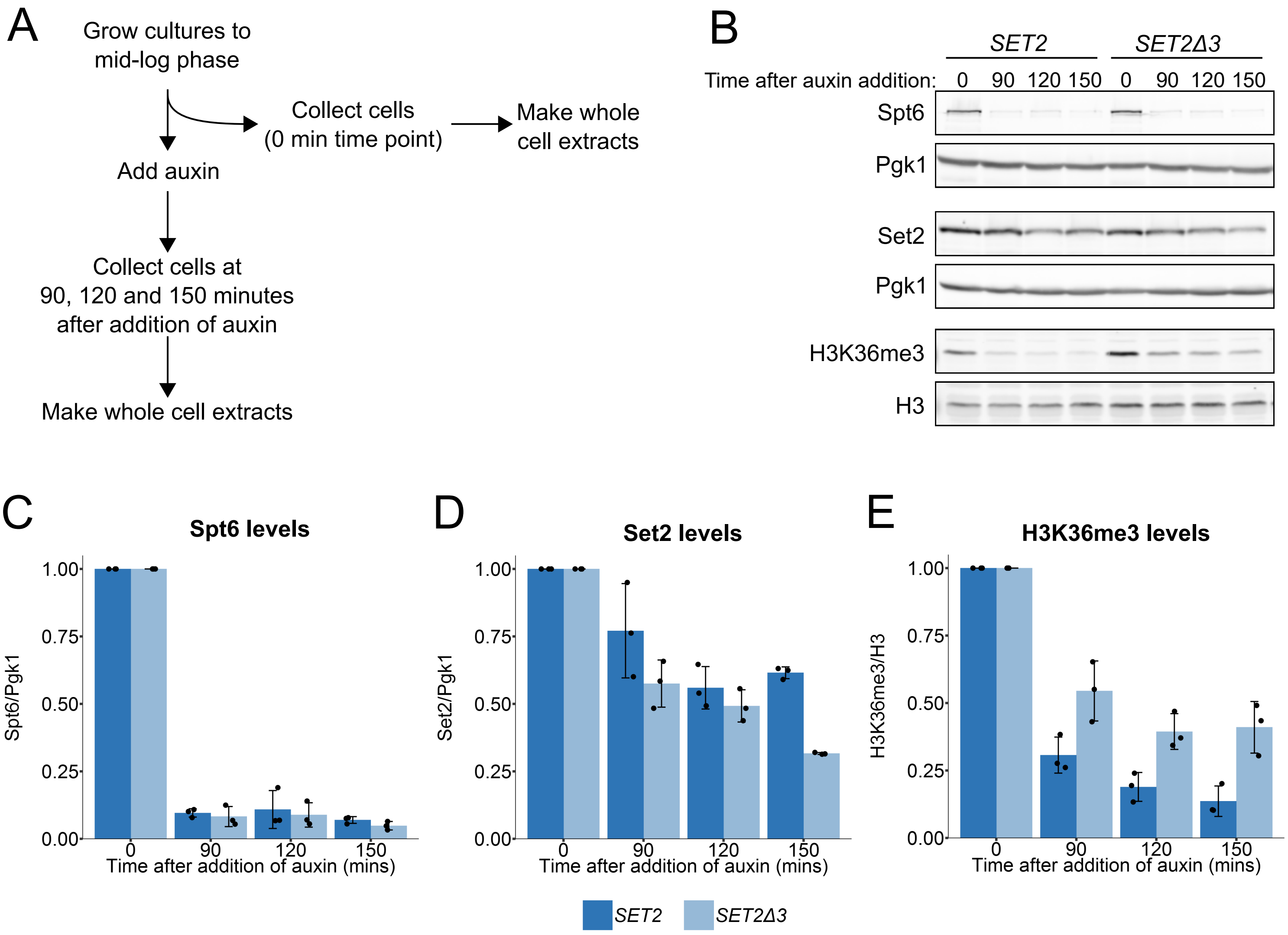

Supplemental Figure S2. *SET2Δ3* cells show reduced loss of H3K36me3 upon Spt6 depletion. (**A**) A schematic illustrating the experimental plan for depletion of Spt6 in wild-type and *SET2Δ3* strains containing a derivative of *SPT6* fused to sequences encoding an auxin-inducible degron. Addition of the auxin IAA promotes degradation of Spt6. (**B**) Western blots assaying Spt6, Set2, and H3K36me3 levels in wild-type and *SET2Δ3* strains upon Spt6 depletion. Pgk1 and histone H3 were used as loading controls. (**C-E**) Quantification of western blots assaying Spt6 (**C**), Set2 (**D**) and H3K36me3 (**E**) levels upon Spt6 depletion, with and without the *SET2Δ3* mutation, normalized to their respective loading controls. The black dots represent the individual data points for three experiments and the bars show the mean +/- standard deviation. Proteins levels for each strain have been normalized to the 0 minute time point.

Supplemental Figure S3

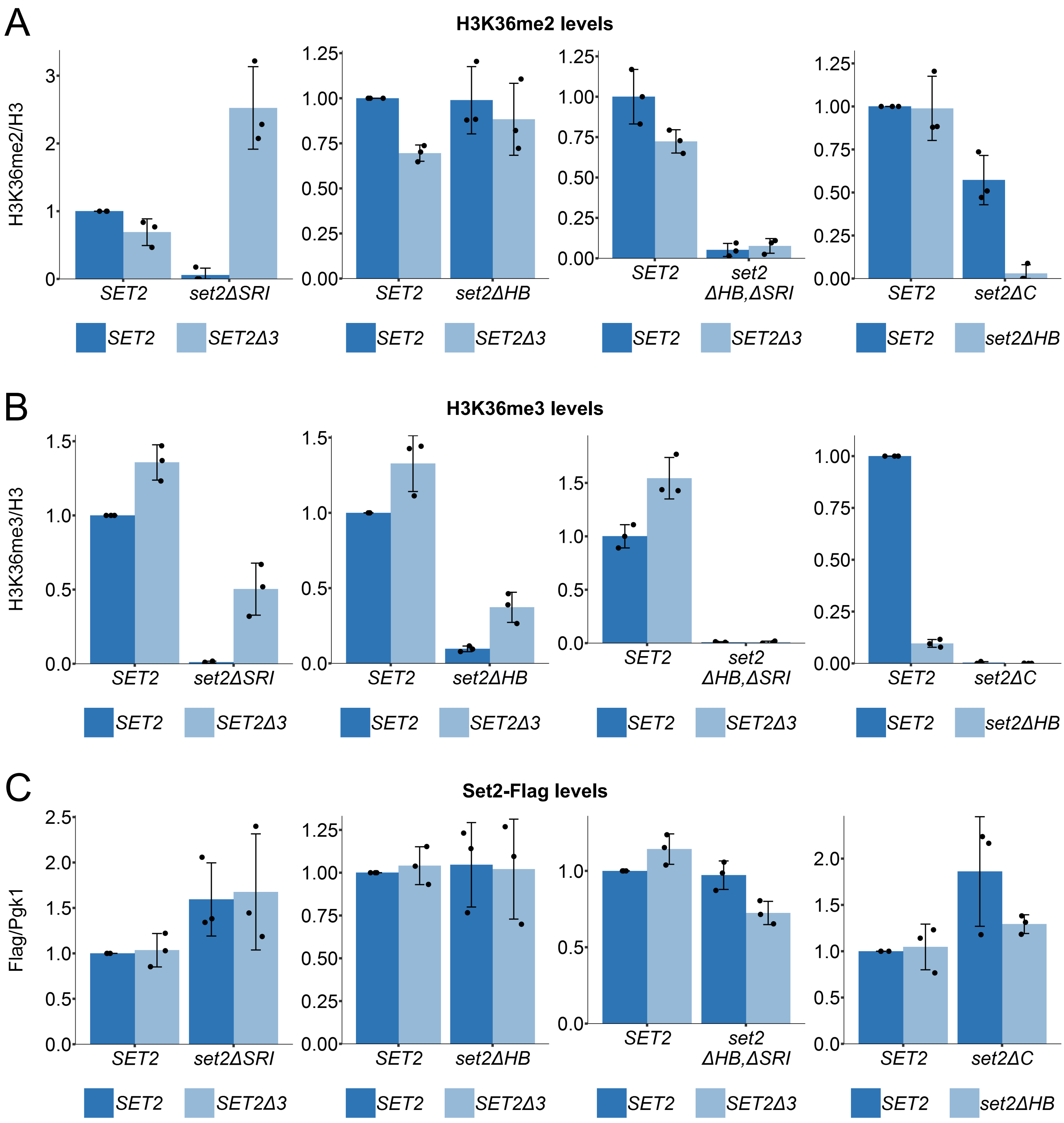

Supplemental Figure S3. *SET2Δ3* rescues H3K36 methylation and intragenic transcription upon deletion of the SRI or HB domains. (**A-C**) Quantification of western blots assaying H3K36me2 (**A**), H3K36me3 (**B**) and FLAG-tagged Set2 levels (**C**) in strains with the indicated *set2* mutations. The black dots represent the individual data points for three experiments and the bars show the mean +/- standard deviation. The plotted values for *SET2*, *SET2Δ3* and *set2ΔHB* are the same between some of the bargraphs, but have been plotted again for ease of representation.

Supplemental Figure S4

A

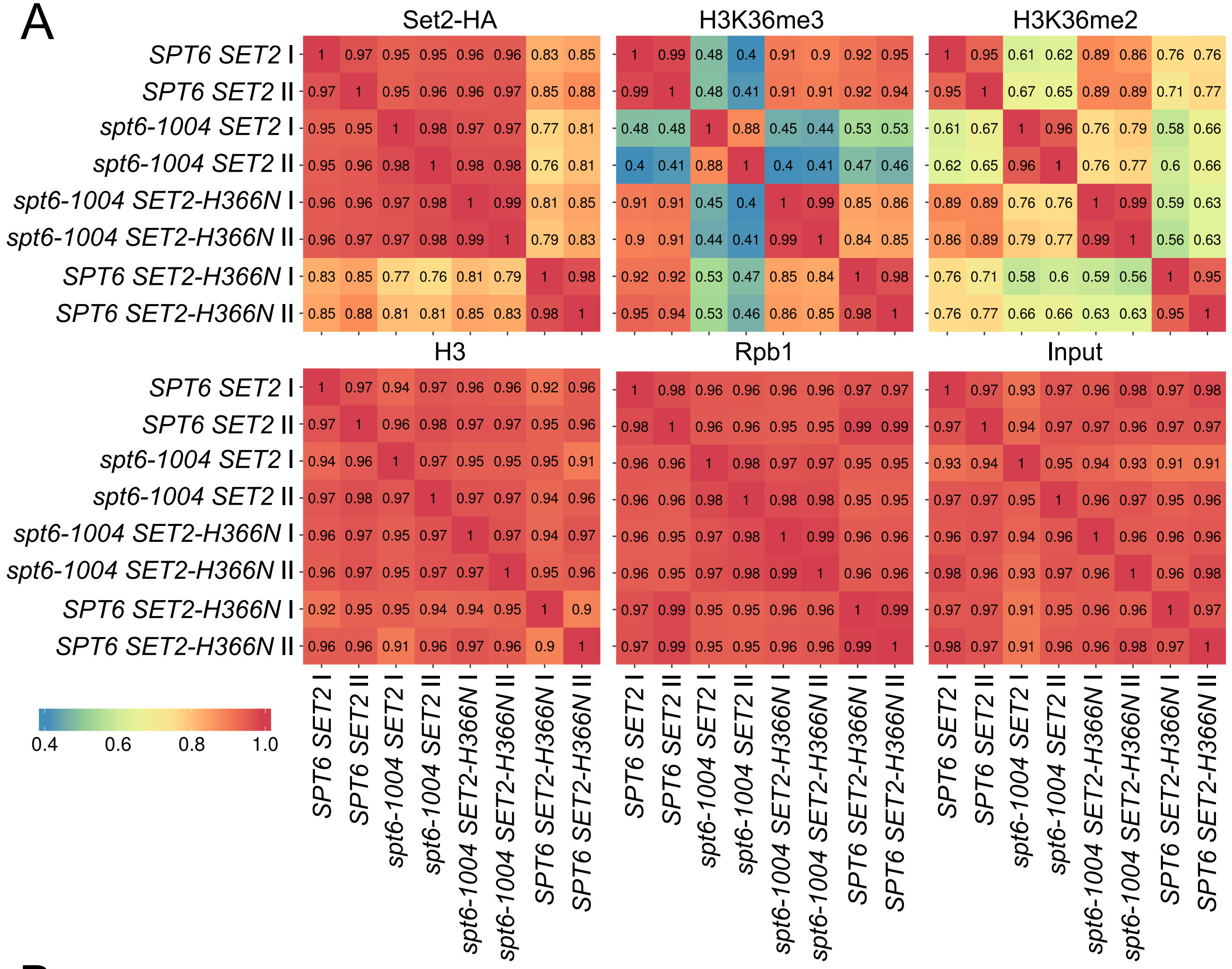

C

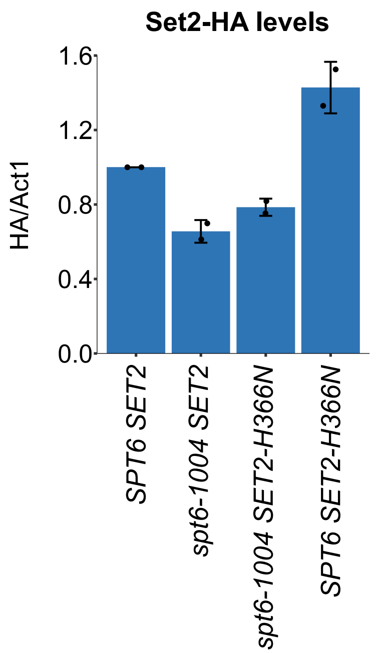

Supplemental Figure S4. Quality analyses of ChIP-Seq data. (**A**) Correlation heatmaps for all 48 samples that were processed for ChIP-Seq. The numbers represent the Pearson correlation coefficient of coverage over 20 bp bins tiling the entire genome. (**B**) Bargraphs showing the proportion of reads in each library mapped to the *S. cerevisiae* or *S. pombe* (spike-in control) genome. (**C**) Quantification of western blots assaying HA tagged Set2 protein levels prepared from the same cultures that were processed for ChIP-Seq. The black dots represent the individual data points for two experiments and the bars show the mean +/- standard deviation.

B

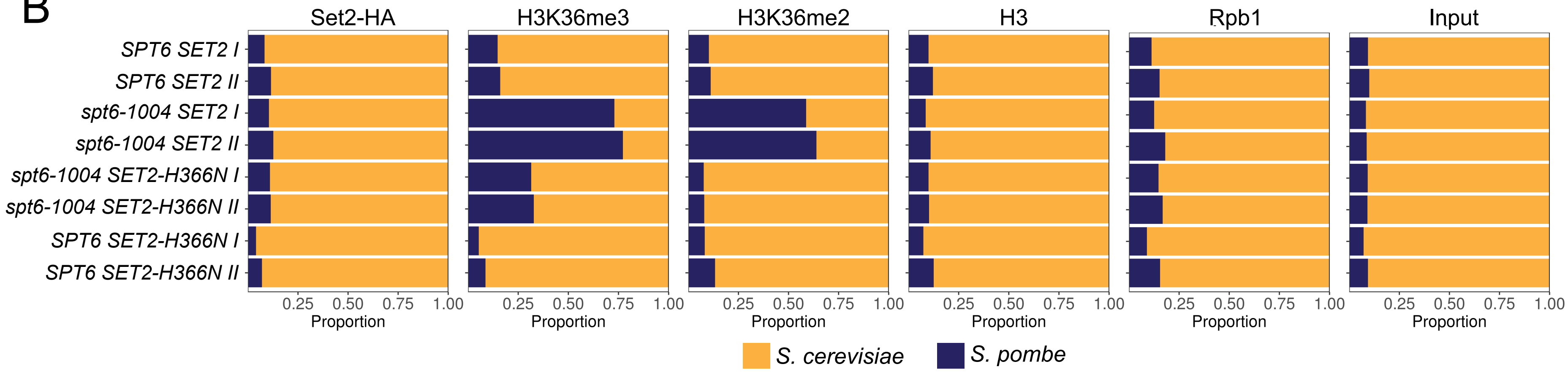

Supplemental Figure S5

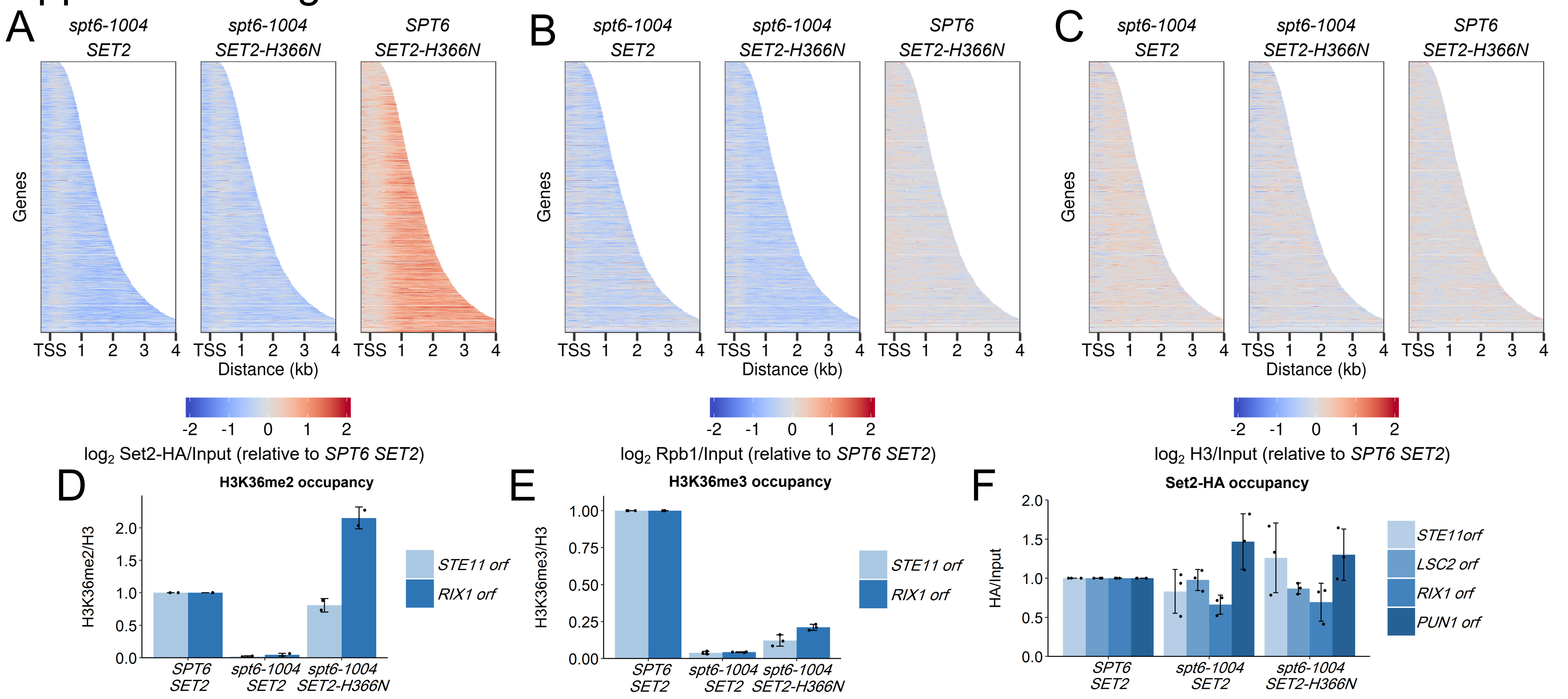

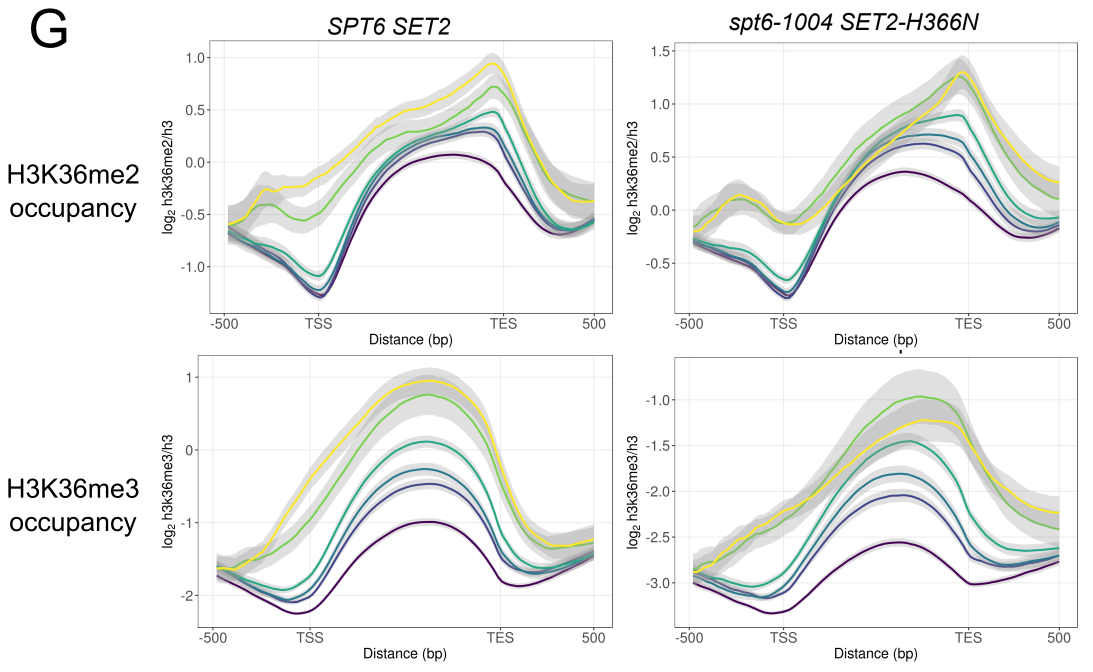

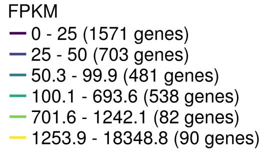

Supplemental Figure S5. Control analyses for ChIP-Seq data. (**A-C**) Heatmaps depicting Set2-HA/Input (**A**), Rpb1/Input (**B**) and H3/Input (**C**) levels for all non-overlapping genes that code for protein (n = 3522). All values that had a log fold change of below -2 or above 2 have been set to -2 or 2 respectively. (**D-F**) ChIP-qPCR analysis quantifying H3K36me2 (**D**), H3K36me3 (**E**) and Set2-HA (**F**) levels over the indicated genes. ChIP-qPCR data has been spike-in normalized to the *S. pombe ACT1* gene. (**G**) Metagene plots showing average H3K36me2/H3 and H3K36me3/H3 levels for groups of genes that are binned by their level of expression (obtained from RNA-seq data; N. I. Reim and F. Winston, unpublished). The shaded region around the lines represents the 95% confidence interval. All data has been normalized to an *S. pombe* spike-in control.

Supplemental Table S1 - Yeast strains

Strain	Genotype	Purpose	Species	Source
FY3129	*MAT**a** his3Δ200 lys2-128δ ura3-52 leu2Δ1 spt6-1004 STE11-CAN1 can1Δ::HygMX HOΔSTE11-TAP::HIS3MX FLO8-URA3*	Parent for genetic selection	*S. cerevisiae*	This study
FY3130	*MATα his3Δ200 lys2-128δ ura3-52 ade8-104 spt6-1004 STE11-CAN1 can1Δ::HygMX HOΔSTE11-TAP::HIS3MX FLO8-URA3*	Parent for genetic selection	*S. cerevisiae*	This study
FY3131	*MAT**a** his3Δ200 lys2-128δ ura3-52 leu2Δ1 spt6-1004 STE11-CAN1 can1Δ::HygMX HOΔSTE11-TAP::HIS3MX*	Figs. 1,2	*S. cerevisiae*	This study
FY3132	*MATα his3Δ200 lys2-128δ ura3-52 can1Δ::HygMX STE11-CAN1 FLO8-URA3 HOΔSTE11-TAP::HIS3MX*	Figs. 1,2	*S. cerevisiae*	This study
FY3133	*MAT**a** his3Δ200 lys2-128δ ura3-52 leu2Δ1 spt6-1004 STE11-CAN1 can1Δ::HygMX HOΔSTE11-TAP::HIS3MX SET2-G367S*	Figs. 1,2	*S. cerevisiae*	This study
FY3134	*MAT**a** his3Δ200 lys2-128δ ura3-52 leu2Δ1 spt6-1004 STE11-CAN1 can1Δ::HygMX HOΔSTE11-TAP::HIS3MX SET2Δ3*	Figs. 1,2	*S. cerevisiae*	This study
FY3135	*MAT**a** his3Δ200 lys2-128δ ura3-52 leu2Δ1 spt6-1004 STE11-CAN1 can1Δ::HygMX HOΔSTE11-TAP::HIS3MX SET2-H366N*	Figs. 1,2	*S. cerevisiae*	This study
FY3136	*MAT**a** his3Δ200 lys2-128δ ura3-52 leu2Δ1 spt6-1004 STE11-CAN1 can1Δ::HygMX HOΔSTE11-TAP::HIS3MX SET2-L365Q*	Figs. 1,2	*S. cerevisiae*	This study
FY3203	*MATa his3Δ200 lys2-128δ ura3-52 set2Δ:: kanMX can1Δ::HygMX FLO8-URA3 STE11-CAN1 HOΔSTE11-TAP-HIS3MX*	Fig. 1	*S. cerevisiae*	This study
FY3204	*MAT**a** his3Δ200 lys2-128δ ura3-52 leu2Δ1 can1Δ::HygMX STE11-CAN1 FLO8-URA3 HOΔSTE11-TAP::HIS3MX spt6-1004 set2Δ::NatMX*	Fig.1	*S. cerevisiae*	This study
FY3137	*MAT*α *his3Δ200 lys2-128δ ura3-52 leu2Δ1 FLO8-URA3*	Figs. 1,2,3,5 and Supplementary Fig. 1	*S. cerevisiae*	This study
FY3138	*MAT*α *his3Δ200 lys2-128δ ura3-52 leu2Δ1 FLO8-URA3 spt6-1004*	Figs. 1,2,3 and Supplementary Fig. 1	*S. cerevisiae*	This study
FY3139	*MAT*α *his3Δ200 lys2-128δ ura3-52 leu2Δ1 FLO8-URA3 SET2Δ3* (first isolate)	Figs. 1,2,3,5	*S. cerevisiae*	This study
FY3140	*MAT*α *his3Δ200 lys2-128δ ura3-52 leu2Δ1 FLO8-URA3 spt6-1004 SET2Δ3* (first isolate)	Figs. 1,2,3 and Supplementary Fig. 1	*S. cerevisiae*	This study
FY3201	*MAT*α *his3Δ200 lys2-128δ ura3-52 FLO8-URA3 leu2Δ1 spt6-1004 SET2Δ3* (second isolate)	Fig. 2	*S. cerevisiae*	This study
FY3202	*MAT*α *his3Δ200 lys2-128δ ura3-52 leu2Δ1 FLO8-URA3 SET2Δ3* (second isolate)	Fig. 2	*S. cerevisiae*	This study
FY3141	*MAT**a** his3Δ200 lys2-128δ ura3-52 leu2Δ1 trp1Δ63 can1Δ::HygMX HOΔSTE11-TAP::HIS3MX STE11-CAN1 spt6-1004 (hht1-hhf1)Δ::kanMX (hht2-hhf2)Δ::natMX [HHT2-HHF2/TRP1/CEN/ARS]*	Fig. 3	*S. cerevisiae*	This study
FY3142	*MAT**a** his3Δ200 lys2-128δ ura3-52 leu2Δ1 trp1Δ63 can1Δ::HygMX HOΔSTE11-TAP::HIS3MX STE11-CAN1 spt6-1004 (hht1-hhf1)Δ::kanMX (hht2-hhf2)Δ::natMX SET2-H366N [HHT2-HHF2/TRP1/CEN/ARS]*	Fig. 3	*S. cerevisiae*	This study
FY3143	*MAT**a** his3Δ200 lys2-128δ ura3-52 leu2Δ1 trp1Δ63 can1Δ::HygMX HOΔSTE11-TAP::HIS3MX STE11-CAN1 spt6-1004 (hht1-hhf1)Δ::kanMX (hht2-hhf2)Δ::natMX [hht2-K36A-HHF2 TRP1 CEN3]*	Fig. 3	*S. cerevisiae*	This study

FY3144	*MAT* **a** *his3Δ200 lys2-128δ ura3-52 leu2Δ1 trp1Δ63 can1Δ::HygMX HOΔSTE11-TAP::HIS3MX STE11-CAN1 spt6-1004 (hht1-hhf1)Δ::kanMX (hht2-hhf2)Δ::natMX SET2-H366N [hht2-K36A-HHF2 TRP1 CEN3]*	Fig. 3	*S. cerevisiae*	This study
FY3145	*MAT* **a** *his3Δ200 lys2-128δ ura3-52 FLO8-URA3 ctk1Δ::NatMX*	Fig. 5	*S. cerevisiae*	This study
FY3146	*MAT* α *his3Δ200 lys2-128δ ura3-52 leu2Δ1 FLO8-URA3 ctk1Δ::NatMX SET2Δ3*	Fig. 5	*S. cerevisiae*	This study
FY3147	*MAT* α *his3Δ200 lys2-128δ ura3-52 FLO8-URA3 paf1Δ::NatMX (second isolate)*	Fig. 5	*S. cerevisiae*	This study
FY3148	*MAT* α *his3Δ200 lys2-128δ ura3-52 leu2Δ1 FLO8-URA3 ctr9Δ::NatMX*	Fig. 5	*S. cerevisiae*	This study
FY3149	*MAT* α *his3Δ200 lys2-128δ ura3-52 leu2Δ1 FLO8-URA3 ctr9Δ::NatMX SET2Δ3*	Fig. 5	*S. cerevisiae*	This study
FY3150	*MAT* α *his3Δ200 lys2-128δ ura3-52 leu2Δ1 FLO8-URA3 paf1Δ::NatMX SET2Δ3*	Fig. 5	*S. cerevisiae*	This study
FY3151	*MAT* **a** *his3Δ200 lys2-128δ ura3-52 FLO8-URA3 ctk1Δ::NatMX SET2Δ3*	Fig. 5	*S. cerevisiae*	This study
FY3152	*MAT* α *his3Δ200 lys2-128δ ura3-52 FLO8-URA3 paf1Δ::NatMX (first isolate)*	Fig. 5	*S. cerevisiae*	This study
FY3153	*MAT* **a** *his3Δ200 lys2-128δ ura3-52 FLO8-URA3 ctr9Δ::NatMX*	Fig. 5	*S. cerevisiae*	This study
FY3154	*MAT* **a** *his3Δ200 lys2-128δ ura3-52 FLO8-URA3 ctr9Δ::NatMX SET2Δ3*	Fig. 5	*S. cerevisiae*	This study
FY3155	*MAT* **a** *his3Δ200 lys2-128δ ura3-52 FLO8-URA3 paf1Δ::NatMX SET2Δ3*	Fig. 5	*S. cerevisiae*	This study
FY3156	*MAT* **a** *his3Δ200 lys2-128δ ura3-52 FLO8-URA3 set2ΔSRI-3xFLAG-NatMX*	Fig. 6 and Supplementary Fig. 3	*S. cerevisiae*	This study
FY3157	*MAT* α *his3Δ200 lys2-128δ ura3-52 leu2Δ1 FLO8-URA3 set2ΔSRI-3xFLAG-NatMX*	Fig. 6 and Supplementary Fig. 3	*S. cerevisiae*	This study
FY3158	*MAT* **a** *his3Δ200 lys2-128δ ura3-52 FLO8-URA3 SET2Δ3,ΔSRI-3xFLAG-NatMX*	Fig. 6 and Supplementary Fig. 3	*S. cerevisiae*	This study
FY3159	*MAT* α *his3Δ200 lys2-128δ ura3-52 leu2Δ1 FLO8-URA3 SET2Δ3,ΔSRI-3xFLAG-NatMX*	Fig. 6 and Supplementary Fig. 3	*S. cerevisiae*	This study
FY3160	*MAT* **a** *his3Δ200 lys2-128δ ura3-52 FLO8-URA3 SET2Δ3-3xFLAG-NatMX*	Fig. 6 and Supplementary Fig. 3	*S. cerevisiae*	This study
FY3161	*MAT* α *his3Δ200 lys2-128δ ura3-52 FLO8-URA3 SET2Δ3-3xFLAG-NatMX*	Fig. 6 and Supplementary Fig. 3	*S. cerevisiae*	This study
FY3162	*MAT* **a** *his3Δ200 lys2-128δ ura3-52 FLO8-URA3 SET2-3xFLAG-NatMX*	Fig. 6 and Supplementary Fig. 3	*S. cerevisiae*	This study
FY3163	*MAT* α *his3Δ200 lys2-128δ ura3-52 leu2Δ1 FLO8-URA3 SET2-3xFLAG-NatMX*	Fig. 6 and Supplementary Fig. 3	*S. cerevisiae*	This study
FY3164	*MAT* **a** *his3Δ200 lys2-128δ ura3-52 FLO8-URA3 set2ΔC-3xFLAG-NatMX*	Fig. 6 and Supplementary Fig. 3	*S. cerevisiae*	This study
FY3165	*MAT* α *his3Δ200 lys2-128δ ura3-52 leu2Δ1 FLO8-URA3 set2ΔC-3xFLAG-NatMX*	Fig. 6 and Supplementary Fig. 3	*S. cerevisiae*	This study

FY3166	*MAT* α *his3Δ200 lys2-128δ ura3-52 set2ΔHB-3xFLAG-NatMX*	Fig. 6 and Supplementary Fig. 3	*S. cerevisiae*	This study
FY3167	*MAT* α *his3Δ200 lys2-128δ ura3-52 set2ΔHB-3xFLAG-NatMX*	Fig. 6 and Supplementary Fig. 3	*S. cerevisiae*	This study
FY3168	*MAT* α *his3Δ200 lys2-128δ ura3-52 SET2Δ3,ΔHB-3xFLAG-NatMX*	Fig. 6 and Supplementary Fig. 3	*S. cerevisiae*	This study
FY3169	*MAT* α *his3Δ200 lys2-128δ ura3-52 SET2Δ3,ΔHB-3xFLAG-NatMX*	Fig. 6 and Supplementary Fig. 3	*S. cerevisiae*	This study
FY3170	*MAT* α *his3Δ200 lys2-128δ ura3-52 FLO8-URA3 set2ΔHB,ΔSRI-3xFLAG-NatMX*	Fig. 6 and Supplementary Fig. 3	*S. cerevisiae*	This study
FY3171	*MAT* α *his3Δ200 lys2-128δ ura3-52 FLO8-URA3 SET2Δ3,ΔHB,ΔSRI-3xFLAG-NatMX*	Fig. 6 and Supplementary Fig. 3	*S. cerevisiae*	This study
FY3172	*MAT* α *his3Δ200 lys2-128δ ura3-52 set2ΔHB,ΔC-3xFLAG-NatMX*	Fig. 6 and Supplementary Fig. 3	*S. cerevisiae*	This study
FY3173	*MAT* α *his3Δ200 lys2-128δ ura3-52 set2ΔHB,ΔC-3xFLAG-NatMX*	Fig. 6 and Supplementary Fig. 3	*S. cerevisiae*	This study
FY3174	*MAT* **a** *his3Δ200 lys2-128δ ura3-52 FLO8-URA3 set2ΔHB-3xFLAG-NatMX*	Fig. 6	*S. cerevisiae*	This study
FY3175	*MAT* α *his3Δ200 lys2-128δ ura3-52 FLO8-URA3 SET2Δ3,ΔHB-3xFLAG-NatMX*	Fig. 6	*S. cerevisiae*	This study
FY3176	*MAT* α *his3Δ200 lys2-128δ ura3-52 set2ΔSRI-3xFLAG-NatMX*	Fig. 6	*S. cerevisiae*	This study
FY3177	*MAT* α *his3Δ200 lys2-128δ ura3-52 SET2-3xFLAG-NatMX*	Fig. 6	*S. cerevisiae*	This study
FY3178	*MAT* α *his3Δ200 lys2-128δ ura3-52 set2ΔSRI,ΔHB-3xFLAG-NatMX*	Fig. 6	*S. cerevisiae*	This study
FY3179	*MAT* α *his3Δ200 lys2-128δ ura3-52 FLO8-URA3 SET2-3xHA-NatMX*	Fig. 7 and Supplementary Figs. 4,5	*S. cerevisiae*	This study
FY3180	*MAT* α *his3Δ200 lys2-128δ ura3-52 FLO8-URA3 SET2-3xHA-NatMX spt6-1004*	Fig. 7 and Supplementary Figs. 4,5	*S. cerevisiae*	This study
FY3181	*MAT* α *his3Δ200 lys2-128δ ura3-52 FLO8-URA3 SET2-H366N-3xHA-NatMX spt6-1004*	Fig. 7 and Supplementary Figs. 4,5	*S. cerevisiae*	This study
FY3182	*MAT* α *his3Δ200 lys2-128δ ura3-52 FLO8-URA3 SET2-H366N-3xHA-NatMX*	Fig. 7 and Supplementary Figs. 4,5	*S. cerevisiae*	This study
FY3183	*MAT* **a** *his3Δ200 lys2-128δ ura3-52 leu2Δ1 FLO8-URA3 SET2Δ11 spt6-1004*	Supplementary Fig. 1	*S. cerevisiae*	This study
FY3184	*MAT* **a** *his3Δ200 lys2-128δ ura3-52 leu2Δ1 FLO8-URA3 SET2Δ12 spt6-1004*	Supplementary Fig. 1	*S. cerevisiae*	This study
FY3185	*MAT* **a** *his3Δ200 lys2-128δ ura3-52 leu2Δ1 FLO8-URA3 SET2Δ13 spt6-1004*	Supplementary Fig. 1	*S. cerevisiae*	This study
FY3186	*MAT* **a** *his3Δ200 lys2-128δ ura3-52 leu2Δ1 FLO8-URA3 SET2Δ14 spt6-1004*	Supplementary Fig. 1	*S. cerevisiae*	This study
FY3187	*MAT* **a** *his3Δ200 lys2-128δ ura3-52 leu2Δ1 FLO8-URA3 SET2Δ15 spt6-1004*	Supplementary Fig. 1	*S. cerevisiae*	This study
FY3188	*MAT* α *his3Δ200 lys2-128δ ura3-52 leu2Δ1 FLO8-URA3 SET2Δ11 spt6-1004*	Supplementary Fig. 1	*S. cerevisiae*	This study

FY3189	*MAT* α *his3Δ200 lys2-128δ ura3-52 leu2Δ1 FLO8-URA3 SET2Δ12 spt6-1004*	Supplementary Fig. 1	*S. cerevisiae*	This study
FY3190	*MAT* α *his3Δ200 lys2-128δ ura3-52 leu2Δ1 FLO8-URA3 SET2Δ13 spt6-1004*	Supplementary Fig. 1	*S. cerevisiae*	This study
FY3191	*MAT* α *his3Δ200 lys2-128δ ura3-52 leu2Δ1 FLO8-URA3 SET2Δ14 spt6-1004*	Supplementary Fig. 1	*S. cerevisiae*	This study
FY3192	*MAT* α *his3Δ200 lys2-128δ ura3-52 leu2Δ1 FLO8-URA3 SET2Δ15 spt6-1004*	Supplementary Fig. 1	*S. cerevisiae*	This study
FY3193	*MAT* **a** *his3Δ200 lys2-128δ ura3-52 LEU2::TIR4 SPT6-V5-AID::KanMX FLO8-URA3*	Supplementary Fig. 2	*S. cerevisiae*	This study (derived from a strain made by Natalia Reim)
FY3194	*MAT* **a** *his3Δ200 lys2-128δ ura3-52 LEU2::TIR4 SPT6-V5-AID::KanMX FLO8-URA3 SET2Δ3*	Supplementary Fig. 2	*S. cerevisiae*	This study (derived from a strain made by Natalia Reim)
FY3195	*MAT* α *his3Δ200 lys2-128δ ura3-52 leu2Δ1 FLO8-URA3 rco1Δ::NatMX*	Fig. 3	*S. cerevisiae*	This study
FY3196	*MAT* α *his3Δ200 lys2-128δ ura3-52 leu2Δ1 FLO8-URA3 rco1Δ::NatMX spt6-1004*	Fig. 3	*S. cerevisiae*	This study
FY3197	*MAT* α *his3Δ200 lys2-128δ ura3-52 leu2Δ1 FLO8-URA3 rco1Δ::NatMX SET2Δ3*	Fig. 3	*S. cerevisiae*	This study
FY3198	*MAT* **a** *his3Δ200 lys2-128δ ura3-52 FLO8-URA3 rco1Δ::NatMX spt6-1004 SET2Δ3*	Fig. 3	*S. cerevisiae*	This study
FY3199	*MATα his3Δ200 lys2-128δ ura3-52 leu2Δ1 FLO8-URA3 set2Δ::NatMX*	*set2Δ* control for westerns and northerns	*S. cerevisiae*	This study
FY3200	*MATα his3Δ200 lys2-128δ ura3-52 leu2Δ1 FLO8-URA3 set2Δ::NatMX spt6-1004*	*set2Δ* control for westerns and northerns	*S. cerevisiae*	This study
FY2731	*MATa set2Δ::kanMX*	*set2Δ* control for westerns	*S. cerevisiae*	Winston Lab
FWP2	*h+ leu1-32 ade6-210*	Fig. 8	*S. pombe*	Winston Lab
FWP562	*h+ leu1-32 ade6-210 spt6-1::NatMX*	Fig. 8	*S. pombe*	This study
FWP563	*h+ leu1-32 ade6-210 lys7-2 ura5-14 spt6-1::NatMX set2Δ3* (first isolate)	Fig. 8	*S. pombe*	This study
FWP564	*h+ leu1-32 ade6-210 lys7-2 ura5-14 spt6-1::NatMX set2Δ3* (second isolate)	Fig. 8	*S. pombe*	This study
FWP565	*h+ leu1-32 ade6-210 set2Δ3*	Fig. 8	*S. pombe*	This study
FWP505	*h- leu1-32 ade6-210 ura4-Δ18 set2Δ::KanMX*	*set2Δ* control for westerns	*S. pombe*	Winston Lab
FWP566	*h- set2-3xHA-NatMX*	Spike in control for ChIP-seq experiments	*S. pombe*	This study
FWP567	*h- ura4-Δ18 leu1-32 ade6-m210 rpb3+::3x-FLAG-NatMx*	Spike in control for ChIP-qPCR experiments in Fig. 4	*S. pombe*	Winston Lab (Ameet Shetty)
FWP568	*h+ ura4-Δ18 leu1-32 ade6-m210 spt5+::3HA-KanMX*	Spike-in control for ChIP-qPCR experiments in Supplementary Fig. 7	*S. pombe*	Winston Lab (Ameet Shetty)

Supplemental Table S2 - Oligos

Name	Gene	Sequence	Purpose	Organism
FO9897	*STE11-pCORE*	CAAAATAAGAGAGCTTCTTTGCAAGGTTCCGTATTCTGGAGAGCTCGTTTTCGACACTGG	FP for replacing *STE11* +1871 - +2154 with *KanMX-URA3*	*S. cerevisiae*
FO9898	*STE11-pCORE*	CACTTTAGTGCCATAAAAAGAATTAATAAGTAGCCCTTTTTCCTTACCATTAAGTTGATC	RP for replacing *STE11* +1871 - +2154 with *KanMX-URA3*	*S. cerevisiae*
FO9901	*CAN1*	CAAAATAAGAGAGCTTCTTTGCAAGGTTCCGTATTCTGGAATGACAAATTCAAAAGAAGACG	FP for replacing *STE11* Δ+1871 - +2154 :: *KanMX-URA3* with *CAN1*	*S. cerevisiae*
FO9902	*CAN1*	CACTTTAGTGCCATAAAAAGAATTAATAAGTAGCCCTTTTCTATGCTACAACATTCCAAAAT	RP for replacing *STE11* Δ+1871 - +2154 :: *KanMX-URA3* with *CAN1*	*S. cerevisiae*
FO10754	*CAN1-pFA6-a*	CTTAACTCCTGTAAAAACAAAAAAAAAAAAAGGCATAGCACGGATCCCCGGGTTAATTAA	FP for deleting *CAN1* with HygMX	*S. cerevisiae*
FO10755	*CAN1-pFA6-a*	ATGGCGTGGAAATGTGATCAAAGGTAATAAAACGTCATATGAATTCGAGCTCGTTTAAAC	RP for deleting *CAN1* with HygMX	*S. cerevisiae*
FO10775	*STE11*	ACAATCTTCATTATACCCAATCGCTGCGTGCTGGAATTATACTGAGCAAGTCCTATATAC	FP for inserting *STE11-TAP::HIS3MX* at the *HO* locus deleting -1400 bp upstream of the *HO* start codon	*S. cerevisiae*
FO10777	*STE11*	ACTTTTATTACATACAACTTTTTAAACTAATATACACATTGAAAGGCCTGTTTCTTCGTG	RP for inserting *STE11-TAP::HIS3MX* at the *HO* locus deleting -1400 bp upstream of the *HO* start codon	*S. cerevisiae*
FO10861	*SET2-pCORE*	TTACTATCGATGATGACTCTCTTCGTCATCAGGCTATCAAGAGCTCGTTTTCGACACTGG	FP for replacing *SET2* (+1092 - +1111) with *URA3-KanMX*	*S. cerevisiae*
FO10862	*SET2-pCORE*	TGAGGTTGTTCCGTGATAAACAATTTAAGCATTTTGCTAATCCTTACCATTAAGTTGATC	RP for replacing *SET2* (+1092 - +1111) with *URA3-KanMX*	*S. cerevisiae*
FO10868	*SET2*	TTACTATCGATGATGACTCTCTTCGTCATCAGGCTATCAAACTAAACGGTTACACCTGTT	FP for making 103,333 C -> A (creating Set2 H366N)	*S. cerevisiae*
FO10869	*SET2*	TGAGGTTGTTCCGTGATAAACAATTTAAGCATTTTGCTAAAACAGGTGTAACCGTTTAGT	RP for making 103,333 C -> A (creating Set2 H366N)	*S. cerevisiae*
FO10870	*SET2*	TTACTATCGATGATGACTCTCTTCGTCATCAGGCTATCAAACAACACGGTTACACCTGTT	FP for making 103,335 T -> A (creating Set2 L365Q)	*S. cerevisiae*
FO10871	*SET2*	TGAGGTTGTTCCGTGATAAACAATTTAAGCATTTTGCTAAAACAGGTGTAACCGTGTTGT	RP for making 103,335 T -> A (creating Set2 L365Q)	*S. cerevisiae*
FO10872	*SET2*	TTACTATCGATGATGACTCTCTTCGTCATCAGGCTATCAAACTACACAGTTACACCTGTT	FP for making 103,330 G -> A (creating Set2 G367S)	*S. cerevisiae*
FO10873	*SET2*	TGAGGTTGTTCCGTGATAAACAATTTAAGCATTTTGCTAAAACAGGTGTAACTGTGTAGT	RP for making 103,330 G -> A (creating Set2 G367S)	*S. cerevisiae*
FO10874	*SET2*	CTTTTTACTATCGATGATGACTCTCTTCGTCATCAGGCTATCAAATACACCTGTTTTAGC	FP for deleting aa 365-367 (creating Set2Δ3)	*S. cerevisiae*
FO10875	*SET2*	CTACCTGAGGTTGTTCCGTGATAAACAATTTAAGCATTTTGCTAAAACAGGTGTATTTGA	RP for deleting aa 365-367 (creating Set2Δ3)	*S. cerevisiae*
FO10908	*HHT1-HHF1-pFA6a*	ATATTTGCTTGTTGTTACCGTTTTCTTAGAATTAGCTAAACGGATCCCCGGGTTAATTAA	FP for deleting *HHT1-HHF1*	*S. cerevisiae*
FO10909	*HHT1-HHF1-pFA6a*	TTTTGTTCGTTTTTTACTAAAACTGATGACAATCAACAAAGAATTCGAGCTCGTTTAAAC	RP for deleting *HHT1-HHF1*	*S. cerevisiae*
FO10910	*HHT2-HHF2-pFA6a*	GAAAATAATTTCAAACACCGATTGTTTAACCACCGATTGTCGGATCCCCGGGTTAATTAA	FP for deleting *HHT2-HHF2*	*S. cerevisiae*

FO10911	*HHT2-HHF2-pFA6a*	AGTCTAAATGCATAGAAAAAAAAAAAATTCCCGC TTTATATGAATTCGAGCTCGTTTAAAC	RP for deleting *HHT2-HHF2*	*S. cerevisiae*
FO9231	*RCO1-pFA6a*	ATAAAAGACACTTCCATTACCATCTGCTAATAAT AATACACGGATCCCCGGGTTAATTAA	FP for deleting *RCO1*	*S. cerevisiae*
FO9232	*RCO1-pFA6a*	TTCACGTTCCTGATTTATTCTTTATGTATGTACG CCGTTTGAATTCGAGCTCGTTTAAAC	RP for deleting *RCO1*	*S. cerevisiae*
FO10899	*CTK1-pFA6a*	AATAACACAGGGACCATACAGCATAAATTATTT GGTAACACGGATCCCCGGGTTAATTAA	FP to delete *CTK1*	*S. cerevisiae*
FO10900	*CTK1-pFA6a*	CTATTTTTTGTGTCTACTTATTTCAATTGGCTAT ATATCCGAATTCGAGCTCGTTTAAAC	RP to delete *CTK1*	*S. cerevisiae*
FO10901	*PAF1-pFA6a*	GTACAATAGAACAGTGCTCATAATAGTATAAAG GGTCACACGGATCCCCGGGTTAATTAA	FP to delete *PAF1*	*S. cerevisiae*
FO10902	*PAF1-pFA6a*	CTACAGGTTTAAAATCAATCTCCCTTCACTTCTC AATATTGAATTCGAGCTCGTTTAAAC	RP to delete *PAF1*	*S. cerevisiae*
FO10903	*CTR9-pFA6a*	ATTGTCTGGTCCATTTGTGTTGAGAGCAAGAAA AAAAAACCGGATCCCCGGGTTAATTAA	FP to delete *CTR9*	*S. cerevisiae*
FO10904	*CTR9-pFA6a*	AAGTTTCTTTAAAAGTCTTGATTCTAACCCTCGC CTCTTCGAATTCGAGCTCGTTTAAAC	RP to delete *CTR9*	*S. cerevisiae*
FO10876	*SET2-pCORE*	TGAAAACGAGAACATAAATATTGAATTTCTTCAA TCATTGGAGCTCGTTTTCGACACTGG	FP for insertion of *URA3* replacing *SET2* (+949-+1140) or (+949-+1044)	*S. cerevisiae*
FO10877	*SET2-pCORE*	CTTCAGTCTCGTTTCCTTTTCCATCTACCTGAG GTTGTTCTCCTTACCATTAAGTTGATC	RP for insertion of *URA3* replacing *SET2* (+949-+1140) or (+1093-+1140)	*S. cerevisiae*
FO10878	*SET2-pCORE*	CCTGATGACGAAGAGAGTCATCATCGATAGTA AAAAGTCTTCCTTACCATTAAGTTGATC	RP for insertion of *URA3* replacing *SET2* (+949-+1044)	*S. cerevisiae*
FO10879	*SET2-pCORE*	ACAGCAAGATAATAAGATAATTGCATCCAAACT CTTGAAGGAGCTCGTTTTCGACACTGG	FP for insertion of *URA3* replacing *SET2* (+1045-+1092)	*S. cerevisiae*
FO10880	*SET2-pCORE*	ACAATTTAAGCATTTTGCTAAAACAGGTGTAAC CGTGTAGTCCTTACCATTAAGTTGATC	RP for insertion of *URA3* replacing *SET2* (+1045-+1092)	*S. cerevisiae*
FO10881	*SET2-pCORE*	TACTATCGATGATGACTCTCTTCGTCATCAGGC TATCAAAGAGCTCGTTTTCGACACTGG	FP for insertion of *URA3* replacing *SET2* (+1093-+1140)	*S. cerevisiae*
FO10882	*SET2-pCORE*	TTACACCTGTTTTAGCAAAATGCTTAAATTGTTT ATCACGGAGCTCGTTTTCGACACTGG	FP for insertion of *URA3* replacing *SET2* (+1141-+1236)	*S. cerevisiae*
FO10883	*SET2-pCORE*	CATTATCAATTTGTGACGACTCAATACCGTTTC GAGTTGTTCCTTACCATTAAGTTGATC	RP for insertion of *URA3* replacing *SET2* (+1141-+1236)	*S. cerevisiae*
FO10884	*SET2*	TAATAAAGAATGAAAACGAGAACATAAATATTG AATTTCTTCAATCATTGGAACAACCTC	FP for deleting *SET2* (+949-+1140) (*SET2Δ11*)	*S. cerevisiae*
FO10885	*SET2*	ATATCATCTTCTTCAGTCTCGTTTCCTTTTCCAT CTACCTGAGGTTGTTCCAATGATTGA	RP for deleting *SET2* (+949-+1140) (*SET2Δ11*)	*S. cerevisiae*
FO10886	*SET2*	TAATAAAGAATGAAAACGAGAACATAAATATTG AATTTCTTCAATCATTGAGACTTTTTA	FP for deleting *SET2* (+949-+1044) (*SET2Δ12*)	*S. cerevisiae*
FO10887	*SET2*	AGTTTGATAGCCTGATGACGAAGAGAGTCATC ATCGATAGTAAAAAGTCTCAATGATTGA	RP for deleting *SET2* (+949-+1044) (*SET2Δ12*)	*S. cerevisiae*
FO10888	*SET2*	GTGTATTACTACAGCAAGATAATAAGATAATTG CATCCAAACTCTTGAAGCTACACGGTT	FP for deleting *SET2* (+1045-+1092) (*SET2Δ13*)	*S. cerevisiae*
FO10889	*SET2*	TCCGTGATAAACAATTTAAGCATTTTGCTAAAA CAGGTGTAACCGTGTAGCTTCAAGAGT	RP for deleting *SET2* (+1045-+1092) (*SET2Δ13*)	*S. cerevisiae*
FO10890	*SET2*	AGAGACTTTTTACTATCGATGATGACTCTCTTC GTCATCAGGCTATCAAAGAACAACCTC	FP for deleting *SET2* (+1093-+1140) (*SET2Δ14*)	*S. cerevisiae*

FO10891	*SET2*	ATATCATCTTCTTCAGTCTCGTTTCCTTTTCCATCTACCTGAGGTTGTTCTTTGATAGCC	RP for deleting *SET2* (+1093-+1140) (*SET2Δ14*)	*S. cerevisiae*
FO10892	*SET2*	AACTACACGGTTACACCTGTTTTAGCAAAATGCTTAAATTGTTTATCACGACAACTCGAA	FP for deleting *SET2* (+1141-+1236) (*SET2Δ15*)	*S. cerevisiae*
FO10893	*SET2*	GTTTTTACTACATTATCAATTTGTGACGACTCAATACCGTTTCGAGTTGTCGTGATAAAC	RP for deleting *SET2* (+1141-+1236) (*SET2Δ15*)	*S. cerevisiae*
FO9227	*SET2-pFA6a*	TCAAACCTTTCTCCTTTCCTGGTTGTTGTTTTACGTGATCCGGATCCCCGGGTTAATTAA	FP for deleting *SET2*	*S. cerevisiae*
FO9228	*SET2-pFA6a*	GAAAACGTGAAACAAGCCCCAAATATGCATGTCTGGTTAAGAATTCGAGCTCGTTTAAAC	RP for deleting *SET2*	*S. cerevisiae*
FO9140	*SET2-pFA6a*	ATCAACAAGGATGTCTTCTCCTCCACCTTCAACATCATCACGGATCCCCGGGTTAATTAA	FP for tagging *SET2* with 3xHA or 3xFLAG	*S. cerevisiae*
FO9141	*SET2-pFA6a*	GAAAACGTGAAACAAGCCCCAAATATGCATGTCTGGTTAAGAATTCGAGCTCGTTTAAAC	RP for tagging *SET2* with 3xHA or 3xFLAG	*S. cerevisiae*
FO8240	*SET2-pFA6a*	CAGTTGTATCGCGTTCTGGCACTGCGACTACAACTCCTACAGATTCACCCAGTATTGGCGAATCCCCGAAAAAAGCTGCTCGGATCCCCGGGTTAATTAA	FP for deleting *S. pombe SET2*	*S. pombe*
FO8241	*SET2-pFA6a*	ACCAGGTATTAAAGACATGCAATATTTGGAACTATAGTATGGTAGACGTTGCTACTTGTCGCAGTTAAAAATACTGGCGAGAATTCGAGCTCGTTTAAAC	RP for deleting *S. pombe SET2*	*S. pombe*
FO10202	*STE11*	CTTTCGTTATCAGGCTAGCAT	FP for *STE11* 5' ChIP-qPCR	*S. cerevisiae*
FO10203	*STE11*	AGGTAAATCGTTGGTCTTTTC	RP for *STE11* 5' ChIP-qPCR	*S. cerevisiae*
FO10204	*STE11*	GCAGTTTTGGGAGTGTTTACT	FP for *STE11* orf ChIP-qPCR	*S. cerevisiae*
FO10205	*STE11*	CATCAACCATCTTTCTGTGAA	RP for *STE11* orf ChIP-qPCR	*S. cerevisiae*
FO9857	*STE11*	AGAGGTGGTCAAACAGACCG	FP for *STE11* 3' ChIP-qPCR	*S. cerevisiae*
FO4211	*STE11*	GGGCACTAGGCCTGTATTGA	RP for *STE11* 3' ChIP-qPCR (also RP for *STE11* 3' northern probe)	*S. cerevisiae*
FO7068	*act1+*	TGCACCTGCCTTTTATGTTG	FP for *S. pombe ACT1* qPCR	*S. pombe*
FO7069	*act1+*	TGGGAACAGTGTGGGTAACA	RP for *S. pombe ACT1* qPCR	*S. pombe*
FO10921	*LSC2*	TAGCGGGGAAGCCAGTATCT	FP for orf qPCR	*S. cerevisiae*
FO10922	*LSC2*	CGGTTTCTTGGTTTGTCTGTCC	RP for orf qPCR	*S. cerevisiae*
FO10923	*RIX1*	AGAAGGAGGTTGAAACGAACGA	FP for orf qPCR	*S. cerevisiae*
FO10924	*RIX1*	TTCGTGTTTGGGTTCAGGGA	RP for orf qPCR	*S. cerevisiae*
FO10925	*PUN1*	TGAAGGTATCGACGGTGCTC	FP for orf qPCR	*S. cerevisiae*
FO10926	*PUN1*	GGATGTTATGCGAGGAGTCCA	RP for orf qPCR	*S. cerevisiae*
FO10894	*SET2-pFA6a*	CAAAAAACTAGTGGAAGCAAAAGAGGCTAAGCGGTTGAAACGGATCCCCGGGTTAATTAA	FP for inserting 3x FLAG tag deleting SRI domain of Set2 (use FO9141 as RP)	*S. cerevisiae*
FO10895	*SET2-pFA6a*	CTACTGTGAGGAGCCAAATTGTATTGGGTTTCTCGGTGGTCGGATCCCCGGGTTAATTAA	FP for inserting 3x FLAG tag after SET domain of Set2 (use FO9141 as RP)	*S. cerevisiae*
FO10927	*SET2-pCORE*	GAATAACAACGCTGAGGGCCATAAACCTCAAAGGCTTTTTGAGCTCGTTTTCGACACTGG	FP for amplifying *URA3-KanMX* to replace Set2 +93 - +117 (HB domain)	*S. cerevisiae*
FO10928	*SET2-pCORE*	CATATATACAGTCATCCAAGTTTTCAAATTTGGTCAATGCTCCTTACCATTAAGTTGATC	RP for amplifying *URA3-KanMX* to replace Set2 +93 - +117 (HB domain)	*S. cerevisiae*
FO10929	*SET2*	AAGAAATACTGAATAACAACGCTGAGGGCCATAAACCTCAAAGGCTTTTTGCATTGACCA	FP for deleting *SET2* +93 - +117 (*set2ΔHB*)	*S. cerevisiae*

FO10930	*SET2*	CTCTTGTTCGCATATATACAGTCATCCAAGTTTT CAAATTTGGTCAATGCAAAAAGCCTT	RP for deleting *SET2* +93 - +117 (*set2ΔHB*)	*S. cerevisiae*
FO10914	*SET2-pUL57*	TAACAAGAAAGTTAATGGAACGCATTTTCTTGA CTTCAGATCCTAGTGTATGCCGTTCGATCATTG CGCTCATTTGGCTTGGTACTGCTG	FP for insertion of *ura5-lys7* replacing pombe *SET2* (+2258 - +2266)	*S. pombe*
FO10915	*SET2-pUL57*	TTTATAGAACGTAGTATAAACTCAATATCAATGC TGAACTTCTTCAGCATCAAACCAAAAATGTTGT AATTACAAGTCGTTCAATGTCTC	RP for insertion of *ura5-lys7* replacing pombe *SET2* (+2258 - +2266)	*S. pombe*
FO10934	*S.pombe SET2*	AGGATGACTTATTAACAAGAAAGTTAATGGAAC GCATTTTCTTGACTTCAGATCCTAGTGTATGCC GTTCGATCATTGCGTACAACATTT	FP to delete pombe *SET2* (+2258 - +2266) to create *set2Δ3*	*S. pombe*
FO10935	*S.pombe SET2*	GATAACATGGTTTTTATAGAACGTAGTATAAACT CAATATCAATGCTGAACTTCTTCAGCATCAAAC CAAAAATGTTGTACGCAATGATC	RP to delete pombe *SET2* (+2258 - +2266) to create *set2Δ3*	*S. pombe*
FO4210	*STE11*	GGATGTCACCAGAGGTGGTC	FP for *STE11* 3' northern probe (use FO4211 as RP)	*S. cerevisiae*
FO1324	*SNR190*	GGCCCTGATGATAATG	FP for *SNR190* northern probe	*S. cerevisiae*
FO1325	*SNR190*	GGCTCAGATCTGCATG	RP for *SNR190* northern probe	*S. cerevisiae*
FO10961	*SET2*	TTTTTGGATCCTCGAAGAACCAAAGTGTGAGTG	FP for cloning full length SET2 into pGEX6p1	*S. cerevisiae*
FO10975	*SET2*	TTTTTCTCGAGTTATGATGATGTTGAAGGTGGA GGA	RP for cloning full length SET2 into pGEX6p1	*S. cerevisiae*
FO10977	*GST-SET2*	GGATTATTCATACCGTCCCAATGTCCCCTATAC TAGGTTA	FP for amplifying Set2 cloned in the pGEX6p1 plasmid for gibson cloning into pFastBac1	*S. cerevisiae*
FO10978	*GST-SET2*	CAAATGTGGTATGGCTGATTTTATGATGATGTT GAAGGTG	RP for amplifying Set2 cloned in the pGEX6p1 plasmid for gibson cloning into pFastBac1	*S. cerevisiae*
FO10979	*pFastBac1*	CACCTTCAACATCATCATAAAATCAGCCATACC ACATTTG	FP for ampifying pFastBac1 backbone for gibson cloning with SET2	Plasmid
FO10980	*pFastBac1*	TAACCTAGTATAGGGGACATTGGGACGGTATG AATAATCC	RP for ampifying pFastBac1 backbone for gibson cloning with SET2	Plasmid

Supplemental Table S3- Plasmids

**Strain**	**Description**	**Host**	**Source**
FB2798	*pGEX6p1-SET2*	DH5α	This study
FB2799	*pGEX6p1-SET2-H366N*	DH5α	This study
FB2800	*pFastBac1-GST-SET2*	DH5α	This study
FB2801	*pFastBac1-GST-SET2-H366N*	DH5α	This study
FB2802	*bMON14272-GST-Set2*	DH10Bac	This study
FB2803	*bMON14272-GST-Set2-H366N*	DH10Bac	This study

Supplemental Table S4 - *SET2* ^{sup} alleles

**Mutation alias**	**Position of Mutation (1=ATG)**	***SET2* mutation**	**Set2 amino acid change**
LF41α	979 - 980	GT -> AA	V327K
LF12a	1006	C -> G	Q336E
LF38α	1049	T -> C	L350P
LF3α	1049	T -> C	L350P
HF3α	1049	T -> C	L350P
LF6a	1076	G -> C	R359P
S9	1094	T -> A	L365Q
LF9α	1094	T -> A	L365Q
LF39α	1094	T -> C	L365P
LF43a	1094 - 1096	TAC -> CCT	L365P, H366Y
S1	1096	C -> A	H366N
S7	1096	C -> T	H366Y
LF4α	1096	C -> T	H366Y
HF9α	1096	C -> T	H366Y
LF7α	1097	A -> C	H366P
HF6a	1098	C -> A	H366Q
LF5a	1099	G -> A	G367S
HF5α	1099	G -> T	G367C
HF11α	1100	G -> A	G367D
S3	1109	G -> T	C370F
